# Polymer-Based Nanosystems—A Versatile Delivery Approach

**DOI:** 10.3390/ma14226812

**Published:** 2021-11-11

**Authors:** Adelina-Gabriela Niculescu, Alexandru Mihai Grumezescu

**Affiliations:** 1 Department of Science and Engineering of Oxide Materials and Nanomaterials, Faculty of Applied Chemistry and Materials Science, Politehnica University of Bucharest, 011061 Bucharest, Romania; niculescu.adelina19@gmail.com; 2 Research Institute of the University of Bucharest—ICUB, University of Bucharest, 050657 Bucharest, Romania; 3 Academy of Romanian Scientists, Ilfov no. 3, 50044 Bucharest, Romania

**Keywords:** polymer-based nanoparticles, drug delivery, targeted delivery, vaccine delivery, vaccine adjuvants, novel nanocarriers

## Abstract

Polymer-based nanoparticles of tailored size, morphology, and surface properties have attracted increasing attention as carriers for drugs, biomolecules, and genes. By protecting the payload from degradation and maintaining sustained and controlled release of the drug, polymeric nanoparticles can reduce drug clearance, increase their cargo’s stability and solubility, prolong its half-life, and ensure optimal concentration at the target site. The inherent immunomodulatory properties of specific polymer nanoparticles, coupled with their drug encapsulation ability, have raised particular interest in vaccine delivery. This paper aims to review current and emerging drug delivery applications of both branched and linear, natural, and synthetic polymer nanostructures, focusing on their role in vaccine development.

## 1. Introduction

The variety and versatility of polymeric materials have drawn increasing scientific interest in their application in diversified fields [1,2,3,4]. In particular, polymer-based nanoparticles were noted to have advantageous properties for biomedical uses [5]. Features such as safety, stability, good solubility, tunable physicochemical characteristics, biocompatibility, and biodegradability have recommended polymeric nanomaterials for use as vehicles for a broad range of drugs, genes, vaccines, and biomolecules [6,7,8].

Either used alone, in blends, or combined with other types of materials, polymer-based nanoparticles can offer protection to the attached cargos, prolong their circulation time, ensure controlled and targeted release, and enhance cellular uptake efficiency [6,9,10,11,12]. Moreover, certain polymers’ innate antimicrobial, antitumor, or immunostimulant properties can amplify therapeutic outcomes of corresponding incorporated drugs and vaccines [13,14,15,16]. Thus, polymer-based nanoparticles can be employed in the prophylaxis and treatment of various infectious, chronic, or genetic diseases [17,18,19]. 

In this regard, the present paper aims to present the natural and synthetic polymers that are most relevant and most commonly used for delivery purposes, further reviewing the recent advances in the delivery of different cargos and focusing, in more detail, on the role of polymers in the development of vaccine formulations. 

## 2. Polymers Used as Nanocarriers

**Figure 1:**
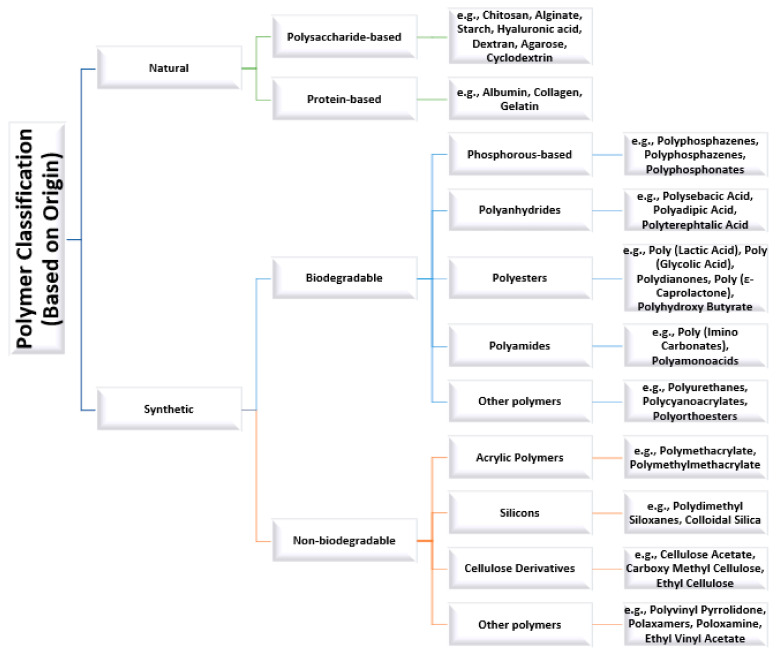
Polymer classification. Created based on information from literature references [11,21,22].

Depending on their origin, two main categories of polymers can be distinguished: natural and synthetic polymers; a more detailed classification is provided in Figure 1. Natural polymers possess superior biocompatibility to synthetic-based materials, as they occur in nature and are fully renewable. In contrast, synthetic polymers are more appealing than natural macromolecular compounds from the reproducibility point of view. Specifically, synthetic polymeric nanomaterials can be produced with negligible inter-batch variation, being engineered with tunable chemical, mechanical, biological, and interfacial properties [11,20]. The features of the most relevant natural and synthetic polymers for delivery applications will be further discussed in more detail. 

### 2.1. Natural Polymers

#### 2.1.1. Chitosan

Chitosan is a highly researched material for polymeric nanocarriers, being a non-toxic, biodegradable, hemocompatible, mucoadhesive polysaccharide generally recognized as safe by the Food and Drug Administration (FDA) [21,23,24,25]. The abundance of hydroxyl and amino groups from its backbone renders this material suitable for chemical modifications and targeted delivery to particular organs or cells [6,26,27]. Moreover, various techniques can be employed for fabricating chitosan drug delivery nanosystems, including ionic gelation, emulsion crosslinking, spray-drying, nanoprecipitation, emulsion solvent diffusion, and reverse micellization method [28,29]. 

The intrinsic antitumor and antimicrobial properties of chitosan have attracted interest in enhancing the efficacy of corresponding loaded substances [23,30]. To put the antitumor potential of this material to use, particular attention has been drawn to the delivery of a plethora of anti-cancer drugs [28] such as doxorubicin [31,32], paclitaxel [33,34,35,36], docetaxel [33,37,38], tamoxifen [39,40], curcumin [40,41,42,43], cisplatin [44,45], and mitomycin C [46,47]. The resulting nanosystems are able to reduce side toxicity while increasing treatment efficiency [28]. Besides, the antimicrobial activity of chitosan can be used in the development of novel antibacterial [48,49,50,51,52,53,54], antifungal [55,56,57,58], and antiviral [59,60,61,62] formulations. 

The mucoadhesive properties of chitosan can be especially exploited for mucosal drug delivery [28]. Chitosan nanoparticles (CSNPs) are suitable for oral and nasal delivery of vaccines and drugs. They produce only small steric obstruction, protect freight therapeutics at the extracellular and intracellular level, prevent rapid clearance, and increase cargo retention time in the mucosa [6,28,63]. CSNPs can also be employed in the ocular delivery of drugs because their hydrophilic nature enhances stability, precorneal retention, and increases interaction with eye mucosa [28,64]. Furthermore, CSNPs can ensure colon-targeted delivery due to their tendency to dissolve in the acidic pH of the stomach and get swollen in the intestinal pH [28]. 

Nonetheless, chitosan is insoluble at physiological pH. However, this drawback can be overcome by chemical modification of the polymer to synthesize novel soluble derivatives [65]. The active functional groups from the structure of chitosan can undergo reactions such as hydroxylation, carboxylation, alkylation, acylation, and esterification that help introduce pendant groups, destroy the crystal structure, and consequently enhance the solubility of the resulted material. This possibility of facile modification expands the chitosan application range and dosage form [23,66,67].

#### 2.1.2. Dextran

The simple and unique features of dextran make it an ideal candidate for nanomedicine carriers. Specifically, this FDA-approved biocompatible and biodegradable bacterial exopolysaccharide is very soluble in water and shows no cytotoxicity after drug delivery [6,68,69]. In contrast to other polysaccharides, dextran is not degraded by salivary amylase or malt amylase, only being broken down by dextranase found in the lumen of the large intestine, liver, spleen, and kidney. Therefore, this material is suitable for encapsulating drugs that must be protected throughout the stomach and small intestine, requiring enhanced absorption of the intestinal epithelium [70]. 

Dextran derivatives (e.g., diethyl aminoethyl (DEAE)-dextran or acetylated dextran (Ac-DEX)) are also promising for delivery applications, demonstrating adjuvant properties, and generating robust immune responses when used as vehicles for vaccine delivery [65,69,71]. 

#### 2.1.3. Alginate

Alginates (also known as sodium-alginates) are a class of unbranched anionic polysaccharides that are attractive for transmucosal administration of drugs due to their mucoadhesive properties [6,21,72]. Moreover, alginate is an FDA-approved polymer that can be orally administered or injected due to its low toxicity, biocompatibility, and biodegradability [73]. In this context, alginate-based nanoparticles were reported as carriers for a broad range of drugs, enzymes, and genes [74].

More recently, alginate biomedical applications were extended to the field of vaccine delivery [75]. Being stable in simulated gastric fluid, alginate nanoparticles can be employed to encapsulate antigens, protecting them from enzymatic degradation and facilitating their release [73]. 

#### 2.1.4. Pullulan

Pullulan is another FDA-approved polysaccharide of interest for bio-nanomedicine. It is non-toxic, non-mutagenic, non-immunogenic, and non-carcinogenic; thus, pullulan-based nanoparticles have important scientific value, receiving great research interest in designing excellent vaccine delivery systems [6,75,76,77]. In addition, pH-sensitive pullulan-based nanocarriers can be employed in anti-angiogenesis and chemotherapy against hepatocellular carcinoma, acting as targeted carriers of genes or proteins without presenting cytotoxic effects to normal cells [75].

Moreover, due to the presence of nine hydroxyl groups in its structure, pullulan can be derivatized to enhance its utility in a broad range of applications, including drug delivery, gene targeting, vaccination, medical imaging, and pharmaceutical dosages formation [75].

#### 2.1.5. Hyaluronic Acid

Hyaluronic acid (HA) is another FDA-approved natural bioadhesive polymer that has been widely investigated for constructing various functional delivery vehicles. Especially due to its mucoadhesive properties, HA can enhance the bioavailability of carried substances through various delivery routes, such as ocular, nasal, and pulmonary administration routes [78,79]. HA-based nanoparticles or nanoconjugates are considered valuable candidates for drug delivery in cancer and atherosclerosis therapy [80]. Delivery effects can be optimized as HA NPs allow selective binding to receptors, such as CD44 and TLR4, which may trigger innate immune responses. Thus, HA site-specific drug delivery systems are valuable options for the targeted release of anticancer drugs and subunit vaccines [6,21,66]. Furthermore, HA structure benefits from ease of chemical modification, extending its application possibilities in drug delivery by conjugating and functionalizing with other molecules [78]. Particularly, their covalent conjugation with lipophilic molecules (e.g., propargylated ferulate fluorophores linked to fatty-acid residues through hexa(ethylene glycol) spacers) represents a promising path for creating self-assembled drug delivery colloidal nanosystems [81]. 

#### 2.1.6. Albumin

Albumin represents an appealing protein-based macromolecular carrier due to its non-toxicity, non-immunogenicity, biocompatibility, easy incorporation of various drugs, and ability to bind with proteins [21,82,83]. Albumin nanoparticles have been shown to exhibit enhanced affinity for anti-cancer drugs, including paclitaxel [84,85,86,87] (FDA-approved nanoformulation as Abraxane^®^/ABI-007 (Celgene) [88]), doxorubicin [87,89,90,91,92], docetaxel [93,94,95,96], tacrolimus [87,97], and curcumin [98,99].

#### 2.1.7. Poly(γ-Glutamic Acid) (γ-PGA)

γ-PGA is a bacterial-produced capsular exopolymer that can be degraded by γ-glutamyl transpeptidase present in the human body. Its advantageous properties, such as low toxicity, non-immunogenicity, biodegradability, and biocompatibility with tissues and cells, have recommended γ-PGA for vaccine development and pharmaceutical applications. Specifically, NPs composed of amphiphilic γ-PGA and hydrophobic amino acids are able to immobilize proteins, peptides, and chemical agents on their surfaces or encapsulate these substances inside the particles [73,100,101].

#### 2.1.8. Other Natural Polymers

β-glucan is another FDA-approved natural polysaccharide of interest for biomedical applications [102]. Particularly, its immunostimulatory properties are appealing for antigen delivery and intensification of the immune response [103]. Moreover, β-glucan can be used as a carrier for targeted drug delivery. Due to its stability, biocompatibility, and specificity, this polymer can be successfully employed in cancer therapies, modulating body immunity in the tumor microenvironment [104,105].

Mannan is also a natural polysaccharide endowed with immunomodulatory properties. When used as a vaccine adjuvant, this polymer can enhance the immune response, especially against the human immunodeficiency virus (HIV) [103]. Other promising applications, for which mannan-based delivery systems have been investigated, include glioblastoma therapy [106], alternative medicine in lung cancer [107], and hypolipidemic medication [108].

The significant hydrophilicity and biocompatibility of cellulose are two main factors that recommend this material for biomedical applications. Recent research demonstrated that cellulose-based hydrogel has cross-sectional porous structures and viscoelastic properties, important features in designing efficient vaccine delivery systems [109]. Specifically, cellulose-based materials have been used as adjuvants for proteins, antigens, or DNA, leading to enhanced immune response [65,110]. Various forms (e.g., nanoparticles, nanowires, or nanofibers) exhibited immunomodulatory properties as they increased the secretion of pro-inflammatory cytokines [65,111,112].

Inulin is a complex natural and hydrophilic polysaccharide useful in the biomedical field, especially due to its unique and flexible structure. Investigations have demonstrated that high molecular weight inulin NPs can be employed to deliver drugs and other molecules of interest. In particular, nanoparticle adjuvants derived from inulin are able to enhance the immune response in vaccines against viruses, such as influenza and hepatitis B [6,75,113].

Other natural polymers that have attracted research interest for delivery purposes include, but are not limited to, glycogen [114,115,116], starch [114,117,118,119,120], lignin [121,122], heparin [123,124], lentinan [14,125,126], and chondroitin sulfate [127,128,129].

### 2.2. Synthetic Polymers 

#### 2.2.1. Polyethyleneimine (PEI)

PEI is an FDA-approved synthetic cationic homopolymer, for human medical applications, that can be used as a transfection reagent or as a material for creating NPs with high nucleic acid complexation capacity [130,131]. PEI has good aqueous solubility and intrinsic pH buffering capacity in the endosomal/lysosomal pathway [132,133]. Due to these favorable features, it can induce endosomal escape of carried agents by the “proton sponge effect”, having a potent mucosal adjuvant activity for viral subunit glycoprotein antigens and promoting antigen cross-presentation [100,130]. However, PEI presents some drawbacks that limit its clinical use. This polymer is not biodegradable, and it exerts toxic effects on cells [130,132]. The toxicity issue can be overcome by conjugating PEI to other polymers, including CS, HA, cyclodextrins, and PEG, to produce safer nanoparticles that can still facilitate endosomal escape [132]. 

#### 2.2.2. Poly (Lactic Acid) (PLA)

PLA is a widely used FDA-approved synthetic polymer in biomedicine, especially for preparing tailored size and shape micro and nanoparticles [6,101,134]. Its versatility, facile synthesis from renewable resources, and biodegradability in extracellular environments have attracted considerable research interest, resulting in numerous investigations for PLA-based drug delivery vehicles [135]. Moreover, this polymer can be chemically altered through interactions with adhesive proteins that endow the material with targeting ability towards specific cells and tissues once it is placed in-situ [136].

#### 2.2.3. Poly (Ethylene Glycol) (PEG)

Another FDA-approved synthetic polymer that gained significant interest for biomedical applications is PEG, especially due to its advantageous properties such as high solubility, non-toxicity, and excellent biocompatibility [11,133,137]. Besides its stand-alone properties, conjugation of PEG to proteins, peptides, and drug delivery systems is a widely employed method for increasing the therapeutic effects of nano-biopharmaceuticals. Known as “PEGylation”, this process endows the nanocarrier with the ability of modulated drug delivery and release [138,139]. However, clinical manifestations of PEG allergy are often severe, imposing attentive consideration concerning its administration and timely diagnosis to prevent anaphylactic reactions [140,141,142,143].

#### 2.2.4. Poly (Lactic-co-Glycolic Acid) (PLGA)

Due to its biodegradability, biocompatibility, and favorable safety profile, PLGA has been approved by the FDA and European Medicines Agency (EMA) for various biomedical applications, including drug and vaccine delivery [65,101,130,144,145]. Moreover, the physicochemical characteristics of PLGA nanosystems can be fine-tuned extensively. This material can also be conjugated with PEG or polyetherimide to form block copolymers that are able to self-assemble into micelles that can encapsulate hydrophobic molecules and hydrophobic peptide antigens or proteins [18]. 

#### 2.2.5. Poly-ε-Caprolactone (PCL)

PCL is one more FDA-approved, biocompatible, and biodegradable synthetic polymer that has attracted attention for nanobiomedicine purposes [146]. Its inexpensiveness, hydrophobicity, stability, and slow degradation pattern are several important features that recommend PCL-based nanoparticles for mucosal antigen delivery and DNA delivery [6,147]. Compared to PLGA, PCL degrades very slowly and without subsequently producing an acidic environment; thus, it is considered a promising adjuvant and carrier candidate for different vaccines [147]. 

#### 2.2.6. Polystyrene (PS)

Despite not being biodegradable, polystyrene nanoparticles (PSNPs) are also attractive for biomedical purposes. PSNPs are biocompatible, do not induce inflammation, bind to a range of antigens due to their easily modifiable surface, and generate CD8+T cell responses specific to the delivered peptides [75,148]. Moreover, PS can be associated with other polymers to create amphiphilic block copolymers that are stable in aqueous media, while also being able to encapsulate hydrophobic bioactive substances [149].

#### 2.2.7. Dendrimers

Dendrimers’ compact, well-defined, highly branched, and radial chemical structure makes this class of synthetic polymers suitable for encapsulating various drugs [6,9]. Bearing multiple surface-accessible functional groups, dendrimers can be employed in coupling with biologically relevant molecules. Moreover, their characteristic three-dimensional structure, size, and surface charge enable them to interact with, and pass through, cell membranes, making them better delivery vehicles than classical polymeric materials [6,130,150]. Nonetheless, the use of dendrimers in biological systems is hindered by their inherent toxicity, mostly attributed to the interaction of surface cationic charge of dendrimers with negatively charged biological membranes [151]. In particular, higher cytotoxicity has been observed for higher-generation dendrimers and for cationic dendrimers, such as poly(amido amine) (PAMAM) and poly(propylene imine) (PPI) [152]. To minimize their toxicity, different chemical modifications can be performed on dendrimers’ surface (e.g., PEGylation, acetylation) [151] or biocompatible molecules (e.g., maltose, maltotriose) can be used to decorate the nanosystem’s outer shell [153].

#### 2.2.8. Other Synthetic Polymers

Phosphazenes are attractive polymers for vaccine formulations. They can induce strong and sustained antigen-specific humoral and cell-mediated immune responses, which are considered better and safer options than conventional adjuvants [75].

Polyanhydrides represent another polymer class of interest for controlled release products. These materials are biodegradable, biocompatible, safe, and approved for human use. Specifically, polyanhydrides degrade through surface erosion, releasing non-toxic and easily metabolized carboxylic by-products. Furthermore, this process of erosion that takes place only at the surface of nanoparticles contributes to the tailored and sustained release of encapsulated cargos [6,65]. Moreover, the surface of polyanhydride particles can be easily functionalized [65].

Polyelectrolytes represent a class of polymers with charged functional groups in their backbone, such as poly(allylamine hydrochloride) (PAH), poly (styrene sulfonate) (PSS), polyacrylic acid (PAA), and poly(diallyl dimethyl ammonium chloride) (PDAC) [154]. Polyelectrolytes can be employed in delivery applications, such as glucose-responsive nanocapsules for protein drug delivery [155], theranostic nanoparticles as MRI-visible drug delivery systems [156], ultrasound-sensitive nanocapsules for remote activated release of biomolecules/drugs [157], and nanocontainers for antibiotic therapy [158].

Polymersomes have attracted increasing research interest as versatile carriers due to their colloidal stability, tunable membrane properties, and capacity of encapsulating various drugs and biomolecules. These vesicles made of self-assembling synthetic block copolymers have tunable stability, degradation, and functionalization. They can deliver hydrophilic compounds by incorporating them inside the vesicle or hydrophobic cargos by membrane delivery [130].

Other synthetic polymers that have attracted research interest for delivery purposes include, but are not limited to, poly β-hydroxybutyrate [159,160], polyurethane (PU) [161,162], polyvinyl pyrrolidone (PVP) [163,164,165], poly (γ-glutamic acid) (PGA) [166,167], and polymethyl methacrylate [168,169,170]. 

## 3. Polymeric Nanoparticles Synthesis

Polymer-based NPs are one of the most commonly used forms of soft materials for nanomedicine applications not only due to their versatility and the broad spectrum of applications but also due to their facile synthesis [88]. Recent polymer chemistry progress has allowed the preparation of tailored NPs with well-controlled structures (e.g., fine-tuned size, shape, morphology) and compositions, which are essential factors in obtaining vehicles for targeted delivery and controlled cargo release [171]. 

**Figure 2:**
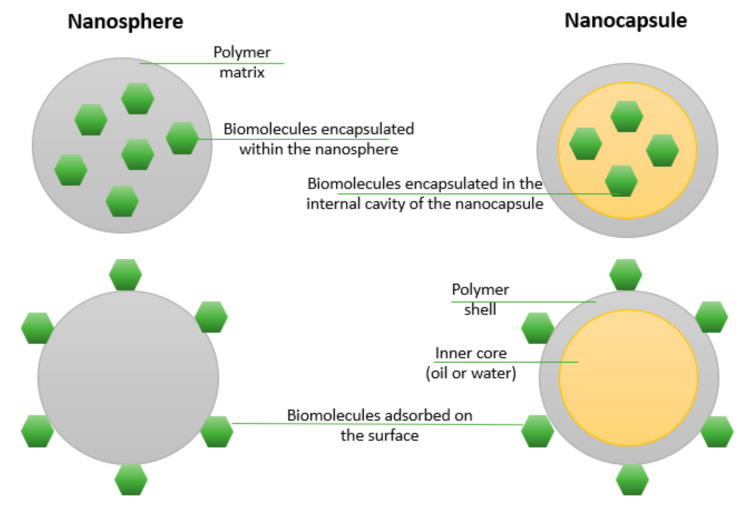
Schematic representation of nanosphere and nanocapsule drug association possibilities. Created based on information from literature references [172,173,174].

In drug delivery applications, two main categories of nanoparticles can be distinguished, namely nanocapsules (reservoir systems) and nanospheres (matrix systems) (Figure 2). Nanocapsules present an inner core in which the freight is usually incorporated, surrounded by a polymeric shell, whereas nanospheres are composed of a continuous polymeric network that can entrap the drug or absorb it onto the nanoparticle’s surface [172]. 

Depending on the type of cargo to be delivered by the polymeric NPs and their proposed administration route, different methods can be employed in the production of nanospheres and nanocapsules [172]. The standard synthesis methods involve one of two fundamental mechanisms: kinetically driven encapsulation, during nucleation and particle growth, and thermodynamically self-assembly. Out of these possibilities, the first one has shown particular promise as it allows the encapsulation of large amounts of hydrophobic drugs while preserving a narrow size distribution [175]. 

**Figure 3:**
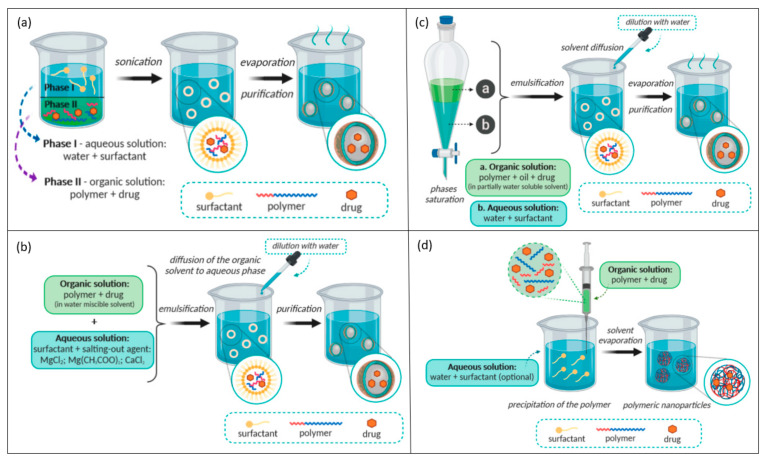
Schematic representation of several polymer nanoparticles synthesis methods: (**a**) solvent evaporation method; (**b**) emulsification/reverse salting-out method; (**c**) emulsification/solvent diffusion method; (**d**) nanoprecipitation method. Reprinted from an open-access source [172].

The first strategy used for manufacturing polymeric NPs from a preformed polymer was the solvent evaporation method (Figure 3a), which leads to the formation of nanospheres. It assumes the preparation of an oil-in-water emulsion, starting from an organic phase (consisting of polar organic solvent, polymer, and drug) and an aqueous phase (consisting of surfactant and water). Initially, dichloromethane and chloroform have been most widely used as organic solvents, but due to toxicity considerations, they have been replaced by ethyl acetate [172,176]. For obtaining small particle size, ultrasonication or high-speed homogenization stages can be employed. This method is suitable for the encapsulation of hydrophobic drugs [177]. A similar synthesis route for nanospheres production is the emulsion/reverse salting method (Figure 3b), which mainly differs from the previous method by the emulsion composition. Specifically, the organic phase is formulated from a polymer, drug, and solvent miscible in water (e.g., acetone, ethanol), and the aqueous phase contains salting-out agents and a stabilizer [176,177]. A derived synthesis method, the emulsification/solvent diffusion technique (Figure 3c), can be used for producing both nanocapsules and nanospheres [177]. This method assumes the formation of an oil-in-water emulsion between a partially water-miscible solvent (e.g., benzyl alcohol, ethyl acetate), containing the polymer and the desired cargo, and an aqueous solution with a surfactant [172]. This method may yield particles with a high encapsulation efficiency of lipophilic and hydrophilic active substances, batch-to-batch reproducibility, narrow size distribution, and ease of scale-up production [176,177]. In contrast to the above-described methods, nanoprecipitation (also known as solvent displacement method or interfacial deposition) (Figure 3d) requires two miscible solvents. The polymer and drug are dissolved in a water-miscible solvent and further injected into an aqueous solution, resulting in a colloidal suspension. The as-such-obtained nanospheres and nanocapsules have a better-defined size, and a narrower size distribution, than the emulsification processes [172,176,177]. 

Other chemical methods for polymeric nanoparticles manufacturing involve the polymerization of monomers, instead of nanoparticles construction, from preformed polymers. In this category, the most used techniques are emulsion polymerization and interfacial polymerization, allowing simultaneous polymer synthesis and drug encapsulation [177]. 

Alternatively, physical methods can be used for polymer NPs manufacturing. One such method is laser ablation, which uses a high-power laser beam to evaporate particles from a solid material source. Similarly, pulse laser deposition (PLD) can be employed; this method assumes that the target material is hit by high-power laser pulses, leading to its melting, evaporation, and ionization. Another technique that provides flexibility and control over surface parameters of the synthesized nanoparticles is electrospraying. The synthesis process starts with a solution of polymer and solvent, placed in a syringe, and the application of a high voltage to its capillary tip. The solvent is evaporated while the particles or fibers are pushed to a collector [178]. 

More recently, polymer-based nanoparticles started being synthesized with the aid of microfluidic devices. The small channel dimensions and the special geometry of these devices allow the synthesis of high-quality nanocarriers in shorter times and with lower consumption of reagents. Moreover, microfluidics technology brings better control over the size, size distribution, morphology, and composition of the final products. Specifically, the size, polydispersity, and drug encapsulation can be simply tailored by varying experimental parameters such as flow rates, polymer composition, and polymer concentration [179]. 

## 4. Applications of Polymer-Based Delivery Nanosystems

Either alone, in blends, or in combination with other nanomaterials, polymer-based nanoparticles can deliver a variety of cargos, including active pharmaceutical ingredients, nucleic acids, imaging agents, antigens, and other biomolecules. This section reviews the most recent advances in the development of polymer-based delivery nanosystems, depending on the carried moieties. 

### 4.1. Drug Delivery

For a drug to be released to the targeted cell, it must be hydrophilic enough to travel through aqueous media and reach the cellular membrane but lipophilic enough to cross this barrier and pass inside the cell. Due to the broad range of available materials and the possibility of functionalization, polymeric materials can be tailored to adjust the hydrophilicity of the drug formulation and deliver the cargo at the desired site. Moreover, the versatility of polymer-based nanoparticles can ensure the delivery of encapsulated drugs through a variety of administration routes, including oral delivery, ocular delivery, nasal delivery, pulmonary delivery, buccal delivery, periodontal delivery, dermal and transdermal delivery, and vaginal delivery.

**Figure 4:**
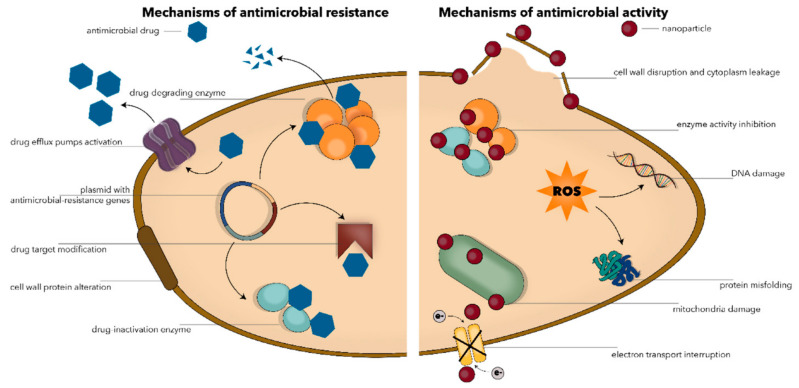
Visual representation of antimicrobial resistance mechanisms (**left**) and antimicrobial activity of nanoparticles (**right**). Reprinted from an open-access source [173].

Given the wide range of possible applications, increasing research interest has been attracted to designing and testing polymer-based delivery platforms. Much effort has recently been put into developing antimicrobial delivery systems that would enhance cargo activity while overcoming drug resistance and diminishing systemic side effects [173,180,181,182,183] (Figure 4). 

Several such novel polymer-based delivery systems are reviewed in Table 1.

**Figure 5:**
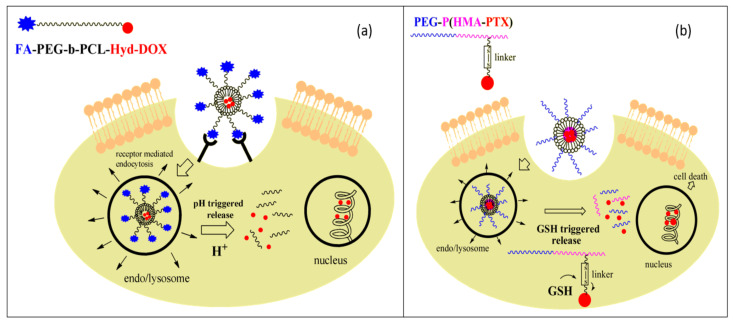
(**a**) Mechanism of action of pH-responsive polymer NPs, decorated with targeting ligand folic acid (FA) and with doxorubicin, bound via a hydrazone bond to diblock copolymer PEG-PCL. (**b**) Mechanism of action of redox-responsive polymer NPs with bonded paciltaxel via a disulfide linker to diblock copolymer PEG-b-PHEMA. Adapted from an open-access source [22].

As cancers remain one of the major health concerns worldwide, extensive research has been oriented to developing better therapeutics for this category of diseases. Chemotherapeutic drugs can be employed in the treatment of cancer patients, as they interfere with the cell cycle and the process of mitosis, causing a greater proportion of cell kill in tumor cells than in healthy tissues [206]. Nonetheless, large systemic doses of such aggressive drugs may lead to drug resistance and adverse effects, while their repeated administration requires a strict treatment schedule that must be adapted to the ability of healthy tissues to recover [207,208,209]. Thus, attention has been drawn to developing carrier systems that allow a controlled release at the tumor site. Due to recent findings concerning the tumor microenvironment, targeted solutions have been envisaged. Specifically, stimuli-responsive delivery systems have been created to target the acidic pH and/or hypoxic environment characteristic of tumor cells [210] (Figure 5). 

In this respect, polymeric nanoparticles have been investigated as carriers towards various tumor cells, including breast [44,211,212,213], colon [91,214,215,216,217], gastric [169,218,219,220], liver [116,221,222], bladder [46,223], skin [224,225], lung [36,92,226], prostate [94,227,228,229], and ovarian [230,231,232] cancer cells (Table 2).

Another attractive and effective cargo for polymeric nanoparticles is represented by photosensitizer drugs and photothermal agents that can be used as adjuvant therapies (e.g., photodynamic therapy, photothermal therapy) in a wide range of diseases [237,238,239,240]. 

Polymer nanoparticles are also useful for delivering drugs to hard-to-reach tissues and organs. For instance, they can facilitate drug permeation to challenging anatomic structures, such as the inner ear [241,242,243,244], retina [245,246,247,248,249], brain [250,251,252,253,254], and avascular connective tissues [255,256,257].

### 4.2. Imaging Agent Delivery

Medical imaging is an essential part of clinical diagnosis, enhancing diagnostic accuracy, enabling a faster start of treatment, and improving survival rates in many diseases [258]. Moreover, synergistic outcomes can be obtained by combining conventional imaging techniques with nanotechnology, especially when using nanoparticles as contrast agents [259,260,261]. Nonetheless, uncoated metal-based nanoparticulate contrast agents may induce toxicological reactions through ROS generation, the release of free metal ions, and the production of aggregates that cannot be eliminated by the cells [261].

Thus, a convenient approach is to coat these NPs with biocompatible polymers. For instance, Vu-Quang et al. [262] designed a nanosystem, based on SPION core covered with a pluronic F127-folate coating, that can specifically target folate receptor-expressing cancer cells—a promising candidate as a contrast agent in MRI. Similarly, Kania et al. [263] have coated SPIONs with ultrathin layers of chitosan derivatives, obtaining suitable T2 contrast agents for liver disease diagnostic. In another study by Amendola et al. [264], bimetallic (silver-iron) nanoparticles were coated with PEG, offering promising results in terms of biopersistency and contrast efficiency. 

Another promising strategy is to deliver conventional contrast agents by polymer-based vehicles. In this respect, Shao et al. [220] have proposed a carboxymethyl chitosan 4-hydroxymethyl-pinacol phenyl borate carrier encapsulated with indocyanine green and modified with RGD. Their ROS-responsive nanosystem can be employed in near-infrared imaging and photothermal therapy against gastric cancer. Another polymer-contrast agent system possibility is offered by Ponsiglione et al. [265], who have delivered Gd-DTPA with the aid of hyaluronic acid. Cheng et al. [266] have also approached Gd delivery using porous polymersomes (produced from self-assembly of polyethylene oxide-*b*-polybutadiene (PBdEO) and polyethylene oxide-*b*-polycaprolactone (PEOCL)). The Gd was conjugated to polyamidoamine (PAMAM) dendrimers via diethylenetriaminepentaacetic acid dianhydride (DTPA dianhydride) before polymersome encapsulation. 

**Figure 6:**
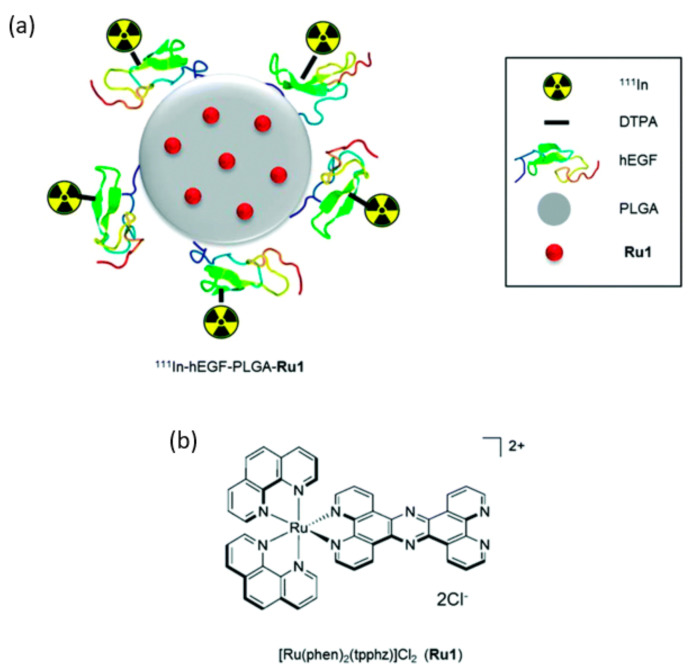
(**a**) Schematic representation of radiolabeled nanoparticles; (**b**) Chemical structure of Ru1. Reprinted from an open-access source [268].

Modern medical imaging can also benefit from polymers tagged with radionuclides for molecular imaging of cancer in techniques such as positron emission tomography (PET) and single-photon emission computed tomography (SPECT) [267]. For instance, Gill et al. [268] have reported the synthesis of PLGA NPs surface conjugated to DTPA-hEGF, encapsulating the ruthenium-based DNA replication inhibitor and radiosensitizer, and labeled with ^111^In (Figure 6). The same radiolabel was used by Gorshkov et al. [269], who conjugated it on N-vinylpyrrolidone-N-vinylformamide copolymers. In a recent study, Huang et al. [270] have prepared ^64^Cu-labelled polymer that can detect small occult tumors in mice’s brain, head, neck, and breast at much higher contrast ^18^F-fluorodeoxyglucose.

### 4.3. Gene Delivery

Gene therapy and immune engineering are complex tasks that hold great promise in treating various disorders. In this respect, nucleic acids can be employed for overexpressing or knocking down specific genes and can be used as adjuvants or danger signals for modulating the behavior of immune cells. Nonetheless, the direct delivery of nucleic acids has several drawbacks, as naked nucleic acids are prone to extracellular degradation, and they face difficulties in passing through the cell membrane [19,271,272].

In this context, increasing research has recently been focused on creating innovative delivery systems that can ensure efficient and targeted delivery of nucleic acids. Among the various tested materials, nanoscale polymers can embed or electrostatically absorb nucleic acids at their surface through a suitable surfactant or cationic polymer addition [6]. Specifically, cationic polymers can form electrostatic nanocomplexes with nucleic acids, which are highly negative, to facilitate their permeation into desired cells. In contrast, other hydrophobic polymers can physically entrap nucleic acids within nanoparticles [19].

Having a positively charged chemical structure, PEI-based nanoparticles are extensively used in gene delivery. However, despite its buffering capacity that can overcome intracellular barriers, PEI use is limited by its toxicity [273,274]. Poly(L-lysine) is another material that has attracted early gene delivery research, as it allows efficient binding to the cargo. Nevertheless, it faces challenges in facilitating endosomal escape and releasing the carried agents inside the cells [19]. 

Currently, lipid-based nanoparticles (LNPs) are the most clinically progressed nanoplatforms for delivering nucleic acids. Nonetheless, Blakney et al. [275] have compared the efficiency of LNP to that of pABOL bioreducible polymer in self-amplifying RNA (saRNA) delivery. Both tested platforms induced enhanced levels of IFN-γ, IL-12, IL-5, and TNF-α 4 h after administration. The researchers obtained a higher humoral and cellular immunity for LNPs, whereas a higher protein expression was observed for pABOL carriers. Thus, each delivery vehicle is advantageous for a different niche of saRNA applications. Specifically, LNPs are more suitable for vaccine formulations, while pABOL nanosystems may be employed in protein replacement therapies.

Another promising approach for nucleic acid delivery is employing lipid-polymer hybrid nanoparticles (LPNs) [276,277,278]. For instance, Vencken et al. [279] have tested the delivery of miR-17 to bronchial epithelial cells by LPNs, composed of PLGA and cationic lipid 1,2-dioleoyloxy-3-(trimethylammonium)propane, noting minimal cytotoxic and pro-inflammatory effects. LPNs can also be employed in gene therapy against drug-resistant glioblastoma, as investigated by Yang et al. [280]. The researchers have recently constructed LPNs loaded with CRISPR/Cas9 plasmids, targeting the MGMT gene, modified with the cRGD peptide that effectively targeted overexpressed integrin αvβ3 receptors in tumor cells, and restored the sensitivity of glioblastoma cells to temozolomide.

### 4.4. Vaccine Delivery 

**Figure 7:**
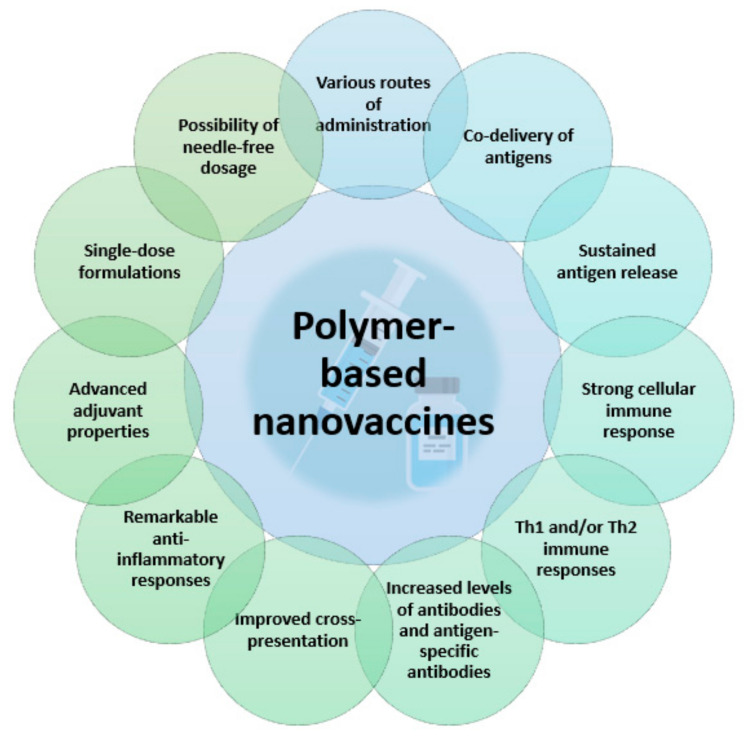
Advantages of polymer-based nanovaccines. Created based on information from literature references [11,18,154,284].

In general, vaccination represents the main method of preventing virus pathogenicity, reducing the burden of many infectious diseases. Nonetheless, traditional vaccines encounter several limitations, as they are susceptible to degradation, have a short duration of action, and may cause side effects and inflammatory reactions at the injection site [6,11]. Moreover, an important number of infectious diseases and chronic disorders (e.g., human immunodeficiency virus (HIV), healthcare-associated infections (HAIs), cytomegalovirus (CMV), respiratory syncytial virus (RSV), tuberculosis, malaria, etc.) cannot be prevented by conventional vaccines [18]. Thus, in recent years, modern bio-nanotechnology started being involved in vaccine development towards creating new-generation formulations [12,281]. In particular, the use of polymer-based nanovaccines is considered a promising approach in improving cross-presentation and enhancing vaccine potency against cancer, intracellular bacteria, and virus infection [282,283]. The main advantages of polymer-based nanovaccines are synthesized in Figure 7.

One attractive approach is to employ polymer nanoparticles, in mucosal delivery of vaccines, as a strategy to overcome some of the drawbacks of conventional vaccines. Such nanovaccines can target both the mucosal and systemic immune systems, enhancing humoral and cell-mediated immune responses, ensuring a sustained release, and protecting the loaded freight against degradation [12]. In more detail, mucosal vaccine delivery may stimulate cytotoxic T-cell responses along with secreted IgA, helping the host organism identify and destroy pathogens before entering further into the body [11]. 

Due to their immunological activity and mucoadhesive properties, CS-based NPs have been widely investigated in developing vaccines against *Clostridium botulinum* type A neurotoxins, *Naospora*, hepatitis B virus, Newcastle disease, and more [6,285]. For instance, Zhao et al. [286] have encapsulated Newcastle disease viruses (NDV) in *N*-2-hydroxypropyl trimethyl ammonium chloride chitosan (N-2-HACC) nanoparticles and assessed their potential as a mucosal immune delivery carrier. The newly developed nanosystems have shown much stronger cellular, humoral, and mucosal immune responses than commercially available live attenuated NDV vaccines.

Another example is offered by Dhakal et al. [287], who have proposed an innovative vaccine delivery platform and tested it against several influenza A virus strains. The researchers evaluated the immune responses and cross-protective efficacy of intranasal administered CSNPs, encapsulated with inactivated SwIAV vaccine, in pigs. The results showed an enhanced IgG serum antibody and mucosal secretory IgA antibody responses in nasal swabs, bronchoalveolar lavage (BAL) fluids, and lung lysates that were reactive against homologous (H1N2), heterologous (H1N1), and heterosubtypic (H3N2) viral strains. Influenza vaccine formulations were also created by use of other bioadhesive polymers [6,65], such as hyaluronic acid [288,289], alginate [290], starch [291], and poly(acrylic acid) [291,292]. 

Another intranasal vaccine delivery system has been developed and investigated by Hamzaoui and Laraba-Djebari [293]. Their study focused on PLGA NPs, loaded with *Cerastes* venom for snake envenomation prevention, and their results confirmed this new nano-formulation represents a potent adjuvant system that improves humoral immune response while protecting against high lethal doses of viper venoms. A similar approach for developing an antivenom vaccine was tackled by Mirzaei et al. [294]. The researchers used CS NPs for loading *Echis carinatus* venom in order to stabilize it. Moreover, the obtained antivenom plasma had a considerably higher potency for neutralizing the venom than conventional delivery systems. 

In an effort to prevent antibiotic-resistant pathogen infections, increasing attention has been drawn to developing antibacterial vaccines [295]. In this respect, various nanoparticle-based vaccines, against several bacteria, have shown promising results (Table 3).

## 5. Role of Polymer-Based NPs in Vaccine Development

Due to their extraordinary versatility, polymers play more than just transporter roles in vaccine formulations. Polymeric nanoparticles may possess the dual capability of being both the adjuvant and delivery vehicle, helping in controlled antigen release, inducing rapid and long-lived immunity, prolonging shelf-life at elevated temperatures, enhancing patient compliance, and enabling the rapid development of vaccines for newly emerging infectious disease viruses [10,65,75,310].

### 5.1. Vaccine Adjuvants

As many antigens are poorly immunogenic, adjuvants are added to vaccine formulations to elicit/potentiate the immune response, offer better protection against pathogens, and diminish the required antigen amount for obtaining immunity [12,100,281]. 

The most currently used adjuvants are aluminum-based (or alum compounds) adjuvants and Freund’s adjuvants. However, despite their relative safety and long history of use, aluminum salts may produce adverse effects, including erythema, nodules, contact hypersensitivity, and granulomas. Other drawbacks of alum adjuvants are the bias towards humoral immunity, the necessity of multiple doses, and incompatibility with many antigens. Freund’s adjuvants also present important disadvantages, as the paraffin oil used for these emulsions causes toxicity issues and produces severe local reactions [65,311,312,313]. Hence, better solutions had to be developed. 

There are two main adjuvants types: antigen delivery systems (or depots) and immunostimulatory agents [130]. Some materials are even able to perform both roles simultaneously.

#### 5.1.1. Antigen Delivery

**Figure 8:**
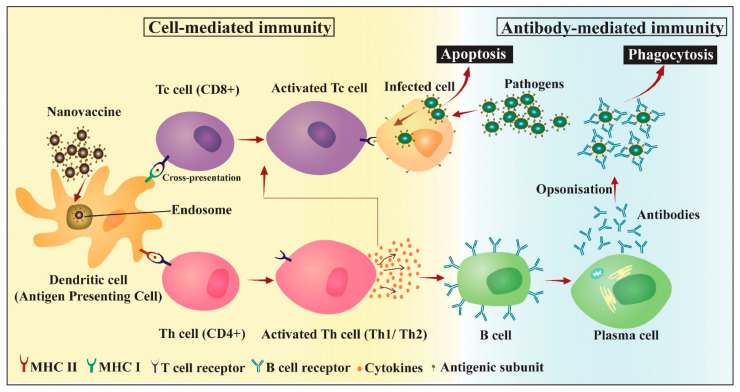
Activation of adaptive immunity by nanovaccines: uptake and presentation of antigenic subunit by APCs elicit cell-mediated and antibody-mediated immune response, leading to apoptosis of infected cells and phagocytosis of antibody–pathogen complex. Reprinted from an open-access source [18].

A variety of polymeric carriers have been investigated for protecting antigens from proteolytic degradation, enhancing antigen entrapment, obtaining a desirable release profile, and targeting antigen-presenting cells (APCs) [144,147,283,314,315,316] (Figure 8). 

**Figure 9:**
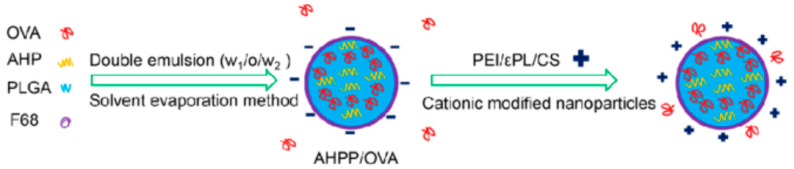
Schematic representation of OVA-loaded surface cationic polymer modified AHPP/OVA nanoparticles. Reprinted from an open-access source [317].

For instance, Wusiman et al. [317] have prepared antigen delivery carriers made of CS-modified PLGA NPs, PEI-modified PLGA NPs, and ε-Poly-L-lysine (εPL)-modified PLGA NPs. The particles were loaded with AHPP and OVA, exhibiting positive charge after surface cationic polymers modification and demonstrating improved antigen loading capacity and stability (Figure 9). Moreover, these formulations allowed greater OVA adsorption capacity, leading to a significantly increased lymphocyte proliferation, improved CD4+/CD8+ T cells ratio, and secretion of cytokines (TNF-α, IFN-γ, IL-4, and IL-6), antibodies (IgG), and antibody subtypes (IgG1 and IgG2a) in immunized mice.

Cruz et al. [318] have also tackled the benefits of PLGA NPs antigen encapsulation. The researchers have co-encapsulated resiquimod and tetanus toxoid peptide antigen in PLGA NPs, obtaining a prolonged controlled release in the endosome. Their findings demonstrated that the slower kinetics of antigen release is more effective for major histocompatibility complex (MHC) class II and I cross-presentation in dendritic cells, producing stronger and more durable immune responses than soluble components. 

By conjugating PLGA with PEG through a peroxalate ester bond and adding PEI as a cationic adjuvant, Liang et al. [319] have synthesized an antigen delivery system that is both ROS responsive and facilitates antigen uptake while diminishing the toxicity associated with cationic adjuvants. The tested nanocarrier proved excellent loading capacity, in vitro stability when encapsulating OVA model antigen, enhanced dendritic cell maturation, improved antigen uptake, increased lysosomal escape, antigen cross-presentation, upregulation of CD4+ and CD8+ T cell proportions, and increased memory T-cell generation.

PLGA has also shown promising results in combination with inorganic materials. In particular, Saengruengrit et al. [320] have reported the successful synthesis of a delivery system based on biocompatible nanocomposite particles of PLGA and superparamagnetic iron oxide nanoparticles (SPIONs). When an external magnetic field was applied, the SPIONs-PLGA system presented superparamagnetic activity, low toxicity, and good uptake in macrophages and bone-marrow-derived primary dendritic cells (BM-DCs). Moreover, the nanodelivery platform did not induce BM-DCs secretion of TNF-α, but it upregulated MHC II, CD80, and CD86 expression and IL-12 and IFN-γ production.

**Figure 10:**
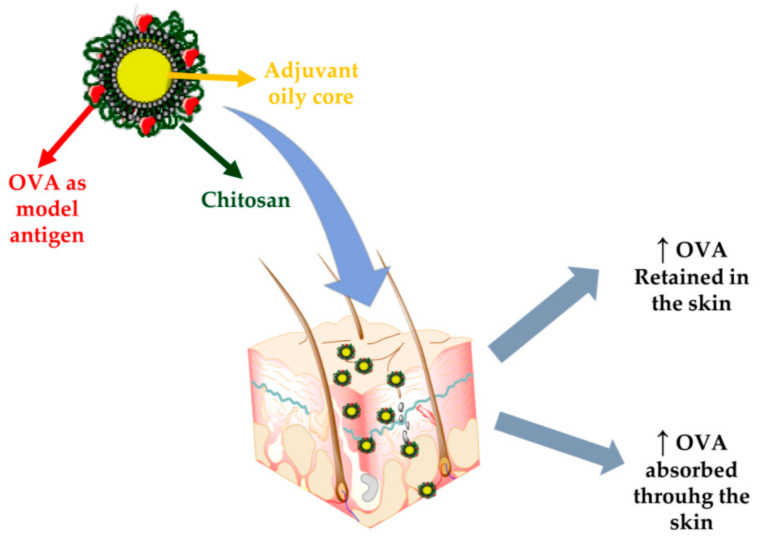
Schematic representation of chitosan-based nanocapsules for transcutaneous antigen delivery. Reprinted from an open-access source [321].

Another widely studied biopolymer for antigen delivery is chitosan. In this respect, Bussio et al. [321] have developed a core-shell structure, with an oily core and a surrounding CS shell of a lower size, for transcutaneous vaccination (Figure 10). CS polymeric corona offered protection to the cargo and exhibited high stability in different storage conditions, along with a significant association of OVA as the model antigen. 

Wang et al. [322] have investigated a system based on polydopamine nanoparticles (Pdop-NPs) for subcutaneous antigen delivery as a vector in cancer immunotherapy. OVA model antigen was grafted onto the nanoparticles to form a carrier system able to migrate to lymph nodes and penetrate APCs. Furthermore, OVA-encapsulated Pdop-NPs promoted the maturation of DCs, activated OVA-specific cytotoxic CD8+ T cells, and induced the production of memory CD4+ and CD8+ T cells, thus considerably suppressing tumor growth.

Another promising delivery system tested for OVA encapsulation is based on lignin nanoparticles. This adjuvant developed by Alqahtani et al. [121] was proven to be a safe stabilizer for antigen formulation during preparation and storage. Moreover, the OVA-encapsulated lignin particles showed no cytotoxicity, significantly higher antigen uptake in dendritic cells, and stronger IgG antibody response than that induced by free OVA alum-adjuvanted OVA, being a potential candidate for the induction of long-term immunity. 

Lipid-polymeric hybrid delivery systems have also started to draw increasing scientific interest. For instance, Miura et al. [323] have created a cholesterol-pullulan self-assembly nanogel that they further modified by carboxylic group substitution to become negatively charged. This innovative system has been shown to target APCs and release the loaded antigen, inducing considerable adaptive immunity. 

#### 5.1.2. Immunomodulation 

One way of enhancing the immune responses is to use a targeted delivery approach to immune cells [324]. In this respect, Dowling et al. [325] have encapsulated a Toll-like receptor (TLR) 8 agonist inside various poly(ethylene glycol)-bl-poly(propylene sulfide) (PEG-bl-PPS) polymer-based nanostructures, allowing direct intracellular release after selective uptake by DCs. TLR 8 agonist polymersomes led to similar newborn DC maturation profiles to those induced by BCG and stronger IL-12p70 production, holding promising potential for early-life immunization against intracellular pathogens. Following a similar strategy for stimulating cellular immunity, Rajput et al. [326] have designed an inulin acetate-based nanodelivery system to target DCs. The tested material exhibited potent vaccine adjuvant properties, activating TLR 4 on multiple immune cells to secrete various cytokines. Widmer et al. [327] proposed a novel carrier nanosystem that can ensure the targeted delivery of resiquimod to the lymph node. The researchers successfully encapsulated this TLR 7 ligand into methoxy poly(ethylene glycol)-b-poly(DL-lactic acid) (mPEG-PLA) and mixed poly(DL-lactic-co-glycolic acid) (PLGA)/mPEG-PLA nanoparticles obtaining good results in terms of cell (i.e., dendritic cells and macrophages) targeting and uptake. Moreover, the investigated particles are non-inflammatory and non-toxic on immune cells, making them promising candidates for cancer immunotherapy.

Another strategy is to take advantage of the intrinsic immunostimulatory properties of certain materials [9,154]. Several polymers, including PLGA, PS, CS, cellulose, lentinan, and dendrimers, can enhance the immune effects of vaccine formulations [14,154,328,329].

The beneficial properties of such polymers can be harnessed for improving the immune response for a broad range of vaccines. For example, inhalable polymeric particles were designed for pulmonary delivery of the hepatitis B vaccine. Thomas et al. [330] have created porous PLGA, as well as PLA NPs loaded with a specific antigen (i.e., HBsAg) that induced enhanced immune responses. Dewangan et al. [331] have also designed an HBsAg PLGA-loaded nanovaccine that demonstrated sustained release and better internalization in macrophage and MRC-5 cell lines. The researchers have tested several single-dose administration routes, obtaining the best results, in terms of immune-stimulating activity, for the intramuscular route; particularly, the nanovaccine administered in this way produced better humoral and cellular responses. An alternative intramuscular delivery system for HBsAg antigen was proposed by Liu et al. [332], who produced PLA microparticles modified with didodecyldimethylammonium bromide that absorbed hepatitis-specific antigens onto their surface. After three intramuscular injections with these particles, the level of pro-inflammatory cytokines (IL-1β, IL-6, CCL2, and CXCL1) increased at the injection site, the vaccine exhibiting ten times higher antigen-specific IgG titers than the group treated with commercial alum-adjuvanted antigen. 

Another vaccine, for which polymers have been shown to potentiate the immune response, is tuberculosis (TB) vaccine. Khademi et al. [333] have combined the vaccine for this disease with chitosan and tested the novel formulation on mice. The CS-based TB vaccine demonstrated how parenteral and non-parenteral immunization lead to appropriate immune responses, inducing both protective and cell-mediated (CD4 and CD8) immune responses in the immunized animal models. Moreover, due to the mucoadhesive properties of CS, non-parenteral immunization can be considered as a more effective administration route. 

Another highly researched topic is the development of an effective HIV vaccine. In this respect, Dacoba et al. [334] have investigated if the covalent attachment of a protease cleavage site (PCS) peptide to polysaccharide-based nanoparticles, together with the administration of polyinosinic:polycytidylic acid, enhanced the immune response. The study obtained promising results, with strong activation of APCs, concluding that both nanoparticle composition and the conjugation of the HIV peptide antigen contributed to the generated humoral and cellular immune responses.

### 5.2. COVID-19 Immunization

As severe acute respiratory syndrome-associated coronavirus 2 (SARS-CoV 2), also known as coronavirus disease of 2019 (COVID-19), has produced a public health crisis worldwide with huge human and economic losses, concerted global efforts have been employed in designing efficient vaccines [335,336,337]. As the genetic sequence of SARS-CoV 2 was made available in record time (within weeks after its discovery), the current vaccines were developed with unprecedented speed, with the clinical trials of promising candidates being completed within only a few months [17,336,338]. 

Nanomedicine played a tremendous role in COVID-19 vaccine development [339]. Moreover, the virus can be regarded as a functional nanomaterial, due to its nanometric size and core-shell nanostructure [340,341]. Thus, various nanoplatforms, such as lipid nanoparticles, polyplexes, dendrimers, cationic polysaccharide particles, and cationic nanoemulsions, were tested for delivering nucleic acids in vaccine formulations [17,275,338,342]. Out of the plethora of possibilities, lipid nanoparticles (LNP) are the most clinically advanced, both Pfizer/BioNTech and Moderna COVID-19 vaccines being LNP formulations [17,275]. 

Nonetheless, polymer-based vaccine alternatives have also shown promising results. For instance, Volpatti et al. [343] have created a subunit nanovaccine by conjugating SARS-CoV-2 Spike protein receptor-binding domain on the surface of polymersomes susceptible to oxidation. This vaccine formulation conducted to strong humoral neutralizing response to SARS-CoV-2 and robust T cell immunity. 

Another strategy was adopted by Zhang et al. [344], who developed a core-shell nanostructure with a core made of PLGA and a human-cell-derived shell sourced from cells that are naturally targeted by SARS-CoV 2. The researchers demonstrated that the virus is neutralized, after incubation with these nanosponges, and can no longer infect cells. 

Polyamidoamines (PAMAMs) represent another promising strategy in the treatment of COVID-19 [340], as it was demonstrated that they could prevent the cleavage of angiotensin and acute respiratory distress syndrome by binding to the ACE2 receptor [345]. Alternatively, chitin and chitosan can be used as delivery vehicles, as they have intrinsic antiviral activities and immune-boosting effects [346]. Other antiviral macromolecules of interest for COVID-19 drugs and vaccines are poly(vinylbenzoic acid), poly(vinylphosphonic acid), PVP, and cyclodextrins [347].

## 6. Conclusions and Future Perspectives

To summarize, a multitude of natural and synthetic polymers can be used to design useful delivery nanosystems for diverse therapeutics, imaging agents, antigens, and other biomolecules. Their versatility and property tunability can be exploited for carrying the necessary moieties to the desired site, even if the cells/tissues are challenging to reach by conventional drugs. Moreover, polymeric nanoparticles allow a targeted and controlled cargo release in response to changes in the pH, the oxygen level in the tissues, or binding with specific receptors. Therefore, polymer-based systems are suitable for many therapies against infections and chronic diseases, offering accurate diagnosis possibilities. This review also explores the role of polymers in developing novel and improved vaccines, especially mucosal administered formulations, for preventing various conditions, including envenomation, hepatitis, tuberculosis, cancer, and COVID-19 infection. 

Considering the recent advances in this field, it can be expected that the particles, experimentally validated on animal models, would move to clinical trials. Nonetheless, further research is required, as a small subset of the immune-activation cascade is usually examined, while overall effects on human health may be neglected. Another challenge that has to be soon overcome is translating from the lab to scale-up synthesis of polymeric nanocarriers without compromising their quality and fine-tuned properties. 

Furthermore, interesting possibilities arise at the convergence of nanotechnology with other innovative fields, such as artificial intelligence and data analytics, that are promising perspectives towards attaining personalized therapeutic and vaccine formulations.

## Figures and Tables

**Table 1:** Polymer-based delivery nanosystems for antimicrobial drugs.

Delivery System	Results	Refs.
**Antimicrobial agent:***Cinnamomum zeylanicum* essential oil **Polymer: Chitosan** **Other materials:** -	Enhanced antibacterial effect compared to free essential oil for all tested bacteria (*Escherichia coli*, *Erwinia carotovora*, and *Pseudomonas fluorescens*) Highest sensitivity was obtained for *P. fluorescens* Maximum antibacterial activity was recorded for *E. coli*	[52]
**Antimicrobial agent:***Origanum syriacum* essential oil **Polymer: Chitosan** **Other materials:** Zn(II)Salen	Good in vitro release profiles Significant growth suppression of microbial species, in the order Gram-positive bacteria > Gram-negative bacteria > fungi	[55]
**Antimicrobial agent:** Nettle essential oil **Polymer: Chitosan** **Other materials:** -	Greater antioxidant activity than free essential oil High antibacterial activity against *Staphylococcus aureus* and *Escherichia coli*	[184]
**Antimicrobial agent:** Clove essential oil **Polymer: Chitosan** **Other materials:** -	Improved antioxidant and antibacterial activities compared to free essential oil High antibacterial activity against *Listeria monocytogenes* and *Staphylococcus aureus*	[185]
**Antimicrobial agent:** N′-((5-nitrofuran-2-yl)methylen)-2-benzhydrazide **Polymer: Chitosan** **Other materials:** Polysorbate, Lyoprotectants (lactose, saccharose, glycine)	Potent antibacterial activity against *Staphylococcus aureus* ATCC 29213, hVISA, and ORSA strains Protective biofilm effect	[51]
**Antimicrobial agent:** Levofloxacin **Polymer: Chitosan** **Other materials:** -	High encapsulation and loading Non-irritant and safe formulation for topical ophthalmic use Strong antibacterial activity against *Pseudomonas aeruginosa* and *Staphylococcus aureus*	[49]
**Antimicrobial agent:** Gentamycin **Polymers: Chitosan, Carbopol 974P** **Other materials:** -	Sustained drug release Safe to the cornea; thus, suitable for ocular delivery Improved patient compliance Antimicrobial susceptibility against *Staphylococcus aureus* and *Escherichia coli*	[186]
**Antimicrobial agent:** Gentamycin **Polymer: Chitosan** **Other materials:** Phosphatidylcholine	Antibiofilm activity through the damaging and removal of pathogens (*Listeria monocytogenes* and *Pseudomonas aeruginosa*) Facilitated antibiotic permeation Neglectable cytotoxicity	[187]
**Antimicrobial agents:** Polyphenol drugs (naringenin, quercetin, curcumin) **Polymers: Chitosan, Dialdehyde cellulose** **Other materials:** L-histidine, Zinc oxide NPs	Sustained drug delivery Potent activity antimicrobial activity against *Staphylococcus aureus* and *Trichophyton rubrum*	[188]
**Antimicrobial agent:** Ampicillin **Polymers: Chitosan, Polyanions** **Other materials:** Phytic acid	High encapsulation efficiency Adequate stability Two-times higher antimicrobial activity than free ampicillin against sensitive and resistant *Staphylococcus aureus* strains	[189]
**Antimicrobial agent:** Silver sulfadiazine **Polymer: Supramolecular polyelectrolyte complexes based on a cyclodextrin-grafted chitosan derivative and carrageenan** **Other materials:** -	Controlled drug delivery (10 times slower drug release than for pure silver sulfadiazine) Strong antibacterial activity against Gram-positive bacteria (*Staphylococcus aureus* and *Enterococcus durans/hirae*) and Gram-negative bacteria (*Klebsiella pneumoniae* and *Escherichia coli*)	[190]
**Antimicrobial agents:** Rifampicin, Ascorbic acid **Polymers: Alginate, Chitosan** **Other materials:** -	Facilitated antibiotic permeation and enhanced cell uptake Significant biocide activity against *Staphylococcus aureus* strains	[191]
**Antimicrobial agent:** LysMR-5 endolysin **Polymers: Alginate, Chitosan** **Other materials:** -	Sustained drug release Biphasic release profile Enhanced bactericidal effect against *Staphylococcus aureus*	[192]
**Antimicrobial agent:** Vancomycin **Polymers: Silk fibroin, Alginate** poly(N-isopropylacrylamide) (PNIPAM) **Other materials:** Growth factor (EGF)	Supported proliferation and growth of fibroblasts Sustained drug release Higher release rate in an alkaline pH compared to neutral pH during 10 days Suitable for severe wound infections	[193]
**Antimicrobial agent:** Vancomycin **Polymer: Hyaluronic acid** **Other materials:** Oleylamine	Sustained drug release for 72 h Moderate antibacterial activity against *Staphylococcus aureus* and methicillin-resistant *S. aureus* (MRSA) 1.8 times higher MRSA cell death than for free drug administration due to a stronger impact on the bacterial membrane	[194]
**Antimicrobial agent:** Triphala Churna (polyherbal formulation) **Polymer: Starch** **Other materials:** -	Excellent antibacterial activity against *Salmonella typhi* and *Shigella dysenteriae* Antibiofilm activity against methicillin-resistant *Staphylococcus aureus* Neuroprotective potential	[117]
**Antimicrobial agent:** SET-M33 peptide **Polymer: Dextran** **Other materials:** -	Effective against *Pseudomonas aeruginosa* Acceptable cytotoxicity Markedly improved lung residence time	[195]
**Antimicrobial agent:** Titanium dioxide **Polymers: Heparin, Polyvinyl alcohol** **Other materials:** -	Good antimicrobial activity against *Staphylococcus aureus* and *Escherichia coli* Improved wound healing Suitable for burn injuries	[196]
**Antimicrobial agent:***Pistacia lentiscus* L. var. *chia* essential oil **Polymer: PLA** **Other materials:** Surfactants (poly(vinyl alcohol—PVA), lecithin—LEC)	Higher encapsulation efficiency was recorded for PLA/PVA NPs than for PLA/LEC NPs A gradual release of the carried agent was noticed for the PLA/PVA NPs, while the PLA/LEC NPs exhibited a more immediate release	[197]
**Antimicrobial agent:** Rifampicin **Polymers: PLA, Poly(L-lysine)** **Other materials:** -	High and superficial loading of the antibiotic Effective delivery with a biphasic release profile Slowed particle migration in the Staphylococcus aureus biofilm thickness Improved retention in the biofilm Better antibiotic efficacy than for uncoated particles	[198]
**Antimicrobial agents:** Rutin, Benzamide **Polymers: PEG, PLGA** **Other materials:** -	Sustained release of rutin-benzamide for several days Antibacterial activity against *Staphylococcus aureus* and *Pseudomonas aeruginosa*Anti-biofilm activity through the disruption of the bacterial membrane and biofilm surface	[199]
**Antimicrobial agent:** Teicoplanin **Polymer: PLGA** **Other materials:** Specific aptamers	Targeted drug delivery There were recorded a 32-fold decrease in minimum concentration values for *Staphylococcus aureus* and a 64-fold decrease for moderately resistant strains, as compared to free teicoplanin	[200]
**Antimicrobial agent:** Red propolis hydroethanolic extract **Polymer: PLGA** **Other materials:** -	96.99% encapsulation efficiency Biofilm inhibitory activity against *Staphylococcus aureus* and *Pseudomonas aeruginosa*	[201]
**Antimicrobial agent:** Farnesol **Polymer: PLGA** **Other materials:** -	Increased irregular cell morphology, membrane and wall damages, and large vacuoles were noted in *Candida albicans* cells Inhibited *Candida* growth and biofilm formation 57% reduced biofilm formation than free farnesol	[202]
**Antimicrobial agents:** Flavonoids (quercetin, rutin) **Polymer: PVP** **Other materials:** -	99.8% entrapment efficiency Higher dissolution rate than unprocessed flavonoids	[203]
**Antimicrobial agent:** Silver nanoparticles **Polymer: PVP** **Other materials:** -	Complete eradication of common otitis media pathogens (i.e., *Streptococcus pneumoniae* and *Haemophilus influenzae*) No in vitro cytotoxicity	[204]
**Antimicrobial agent:** N-diazeniumdiolates (NONOates) **Polymer: Poly(oligo(ethylene glycol)methyl ether methacrylate) (POEGMA)** **Other materials:** Glycidyl methacrylate (GMA)	*Pseudomonas aeruginosa* biofilm dispersal Worm-like particles are more effective in the long term; spherical NPs are better for faster delivery applications	[205]

**Table 2:** Polymer-based delivery nanosystems for chemotherapeutic agents.

Delivery System	Results	Refs.
**Chemotherapeutic agent:** Mitomycin C **Polymer: Chitosan** **Other materials:** Mn:ZnS quantum dots	Diffusion mediated drug release Efficient targeted drug delivery to cancer sites Sustained drug release Effective drug delivery system for non-muscle invasive bladder cancer	[46]
**Chemotherapeutic agent:** Paclitaxel **Polymer: Chitosan** **Other materials:** Polystyrene templates	Sustained drug release Good bioavailability Marked inhibition of lung cancer cells proliferation Promoted apoptosis of cancer cells	[36]
**Chemotherapeutic agent:** Cisplatin **Polymer: Chitosan** **Other materials:** Silver nanoparticles	High encapsulation efficiency Specificity towards breast cancer cells 80% cancer cell death at less than 10 μg doses Minimal cytotoxicity towards healthy cells	[44]
**Chemotherapeutic agent:** Curcumin **Polymer: Chitosan** **Other materials:** -	High encapsulation efficiency The drug was vastly released in the first 5 h, then gradually release up to 90 h Most cancer cells entered apoptosis phase after 72 h of treatment with 150 μM of the drug-carrier system	[43]
**Chemotherapeutic agents:** Tamoxifen, Curcumin **Polymer: Chitosan** **Other materials:** Lipid	High encapsulation efficiency High antioxidant effects Inhibitory activity in the proliferation, growth, and migration of cancer cells	[40]
**Chemotherapeutic agent: *Helianthus tuberosus*** extracts **Polymer: Starch** **Other materials:** Copper oxide NPs, Folic acid	High cytotoxicity to human breast cancer cells due to ROS generation, nuclear damage, and reduction in mitochondrial membrane potential Activation of apoptosis-related protein expression Increased penetration in target cells leads to enhanced breast cancer therapy	[212]
**Chemotherapeutic agent:** Betulinic acid **Polymers: Cellulose, Polymethyl methacrylate** **Other materials:** -	High drug loading capacity Slow drug release rate Satisfactory antitumor activity both in vitro and in vivo Improved cancer cell cytotoxicity Reduced side-effects risk	[168]
**Chemotherapeutic agents:** Doxorubicin, Paclitaxel **Polymer: Alginate** **Other materials:** Oleic acid, Fe_3_O_4_	Increased stability and biocompatibility of the drug-loaded nanocarrier Faster drug release in the acidic medium than in a neutral medium Higher toxicity toward MCF-7 and HeLa cells than free drugs	[233]
**Chemotherapeutic agent:** Doxorubicin **Polymers: Cellulose, Polyacrylamide** **Other materials:** Carboxymethyl-β-cyclodextrin, Folic acid	pH-dependent release behavior Targeted drug release High internalization of cellulose-based NPs lead to fast cellular uptake Reduced dose of doxorubicin and subsequently reduced systemic toxicity	[234]
**Chemotherapeutic agent:** Doxorubicin **Polymer: Lentinan** **Other materials:** -	pH-responsive drug release Enhanced anticancer effects in breast cancer cells Decreased toxicity against healthy cells	[125]
**Chemotherapeutic agent:** Doxorubicin **Polymers: Glycogen, Polypyrrole** **Other materials:** Phospholipids	Efficient specificity and enrichment of hepatocellular carcinoma Controllable drug release to induce cell nucleus damage Synergistic results in combination with photothermal therapy Reduced systemic toxicity Efficient suppression of tumor growth	[116]
**Chemotherapeutic agent:** Doxorubicin **Polymer: Albumin** **Other materials:** -	Drug activity was suppressed under physiological pH, but, in the presence of proteolytic enzymes, 40% of the encapsulated doxorubicin was released from the particles Reduced the metabolic activity of lung carcinoma cells after 72 h Up to 98% cell uptake in cancer cell lysosomal compartment	[92]
**Chemotherapeutic agent:** Doxorubicin **Polymer: Albumin** **Other materials:** -	Cytotoxicity in colon 26 cancer cultures More pronounced *in vivo* anti-tumor activity than free drug Suppression of metastasis	[91]
**Chemotherapeutic agent:** Docetaxel **Polymer: Albumin** **Other materials:** ^131^I	80% of the drug was released at pH 7.4, whereas 93% of docetaxel was released at pH 5.8 Accumulation of drug-carrier system in tumor cells Suitable agent for nuclear imaging and radiotherapy of prostate cancer	[94]
**Chemotherapeutic agent:** Docetaxel **Polymer: Albumin** **Other materials:** Nucleolin-targeted aptamers	Sustained drug release Preferential uptake in nucleolin-expressing CT26 colon cancer cells Enhanced antitumor efficacy compared to non-targeted drug delivery Prolonged survival of the CT26-bearing mice	[96]
**Chemotherapeutic agent:** Docetaxel **Polymer: Albumin** **Other materials:** -	Higher permeability than free drug Controlled drug release Similar cytotoxicity against A549 cells to free drug Lower systemic toxicity than solvent formulated docetaxel	[93]
**Chemotherapeutic agent:** Curcumin **Polymer: Albumin** **Other materials:**	Redox-responsive system and acidic pH-triggered controlled delivery Significantly accelerated drug release in the presence of glutathione Enhanced cellular uptake in MCF-7 cells resulting in higher anticancer efficacy	[98]
**Chemotherapeutic agent:** Paclitaxel **Polymer: Chondroitin sulfate** **Other materials:** Quercetin, Chlorin e6	Redox-responsive system that allows controlled delivery Synergistic results in combination with photodynamic therapy Effective *in vivo* multidrug resistance inhibition and anti-metastasis efficacy	[129]
**Chemotherapeutic agent:** Docetaxel **Polymer: Chondroitin sulfate** **Other materials:** Alpha-tocopherol succinate (TOS), Cystamine	Redox-responsive system that allows controlled delivery Time-dependent qualitative and quantitative uptake by melanoma cells Safe carrier system Enhanced antitumor activity as the drug was delivered accurately, quickly, and thoroughly	[128]
**Chemotherapeutic agent:** Docetaxel **Polymers: PCL, Pluronic F108** **Other materials:** Near infrared dye	Diffusion mediated drug release Increased accumulation of NPs in breast cancer cells Superior targeted drug delivery system	[213]
**Chemotherapeutic agent:** Paclitaxel **Polymers: PLGA, Chitosan** **Other materials:** -	Sustained drug release Faster drug release at pH 5.5 than at pH 7.4 Chitosan modification of PLGA NPs leads to increased cellular uptake and cancer cell viability reduction	[235]
**Chemotherapeutic agents:** Curcumin, Niclosamide **Polymer: PLGA** **Other materials:** -	Much higher drug release at acidic pH 6.0 than at healthy pH of 7.4 Dual drug-loaded particles exhibited a higher anticancer effect than the bare mixture of drugs without PLGA Effective drug-carrier system for MDA-MB-231 breast cancer cells	[236]
**Chemotherapeutic agent:** Doxorubicin **Polymer: PVP** **Other materials:** Gold nanoparticles	Enhanced inhibition of lung cancer cells growth compared to free drug Increased ROS generation Sensitized mitochondrial membrane potential Induced both early and late apoptosis in lung cancer cells Highly upregulated expression of tumor suppressor genes	[226]
**Chemotherapeutic agents:** Quercetin, Gefitinib **Polymer: PVP** **Other materials:** Graphene oxide	Acceptable biocompatibility Efficient drug loading Improved drug release Significantly more toxic than individual drug-loaded systems and free drugs toward PA-1 ovarian cancer cells compared to the toxicity toward IOSE-364 cells	[230]
**Chemotherapeutic agent:** Doxorubicin **Polymers: PMMA, Ovalbumin (OVA)** **Other materials:** Graphene oxide	Successful loading and controlled drug release Higher swelling ratio of the carrier in acidic medium resulting in increased delivery of the drug at pH 2.8 than at normal pH Anti-cancer effect on gastric cancer cells	[169]

**Table 3:** Examples of antibacterial vaccines comprising polymers in their formulation.

Pathogen	Vaccine Formulation	Results	Refs.
*Salmonella*	**Polymer: Chitosan** **Other materials:** Immunogenic outer membrane proteins (OMPs), Flagellin protein	Upregulation of TLRs, and Th1 and Th2 cytokines mRNA expression Enhanced specific systemic IgY and mucosal IgA antibodies responses Reduced *Salmonella* load in the intestines	[296]
*Salmonella*	**Polymer: Chitosan** **Other materials:** OMPs, Flagellin protein	Increased expression of TLR 2, TLR 4, IFN-γ, TGF-β, and Il-4 mRNA expression in chicken cecal tonsils Significantly higher OMPs-specific mucosal IgA production Enhanced lymphocyte proliferation response	[297]
*Salmonella*	**Polymer: Poly (lactic acid)** **Other materials:** Vi polysaccharide and r-flagellin of *Salmonella typhi*	Generated a strong immune response Promoted antibody class switching Produced memory antibody response from single point immunization Enhanced secretion of pro-inflammatory cytokine TNF-α and IL-6, while decreasing IFN-γ production	[298]
*Streptococcus pyogenes*	**Polymers: α-Poly-(L-glutamic acid), Trimethyl chitosan (TMC)** **Other materials:** Peptide antigen	Higher systemic and mucosal antibody titers than antigen adjuvanted with standard mucosal adjuvant cholera toxin B subunit or antigen mixed with TMC Reduced bacterial burden in nasal secretions, pharyngeal surface, and nasopharyngeal-associated lymphoid tissue	[299]
*Streptococcus pyogenes*	**Polymer: Polyacrylate ester-based dendritic polymer** **Other materials:** J14 peptide	Opsonization of pathogen Self-adjuvanting potential	[300]
*Streptococcus pyogenes*	**Polymer: Poly (methyl acrylate)** **Other materials:** B-cell epitope J8, universal T-helper Pan HLA-DR-binding epitope peptide	Strong systemic and mucosal immune responses after a single low-dose immunization Opsonization of pathogen after a second immunization	[301]
*Streptococcus pyogenes*	**Polymers: Polyelectrolyte complexes various formulations, including alginate, chondroitin sulfate, dextran, hyaluronic acid or heparin, TMC** **Other materials:** Liposomes	Anionic polymers assisted in eliciting immune responses while also working as complexing agents PEC-heparin system induced higher antigen-specific systemic IgG and mucosal IgA titers than all other tested PECs	[302]
*Streptococcus pyogenes*	**Polymer: Polyethyleneimine** **Other materials:** Liposomes Lipidated B-cell epitope, T-helper epitope	Significant mucosal and systemic immunity Production of IgA and IgG antibodies	[303]
*Streptococcus pneumoniae*	**Polymer: Polymeric caffeic acid** **Other materials:** Pneumococcal surface protein A (PspA)	Induction of PspA-specific antibody responses in the mucosal and systemic compartments Intranasal vaccination resulted in antigen-dependent protective immunity against a lethal infection of the pathogen	[304]
*Streptococcus agalactiae*	**Polymer: Poly(lactic-co-glycolic acid)** **Other materials:** CAMP factor	Induced a sustained increase od antibody titers Mortality and bacteria counts were lower than in the control group No pathological lesions were detected	[305]
*Pseudomonas aeruginosa*	**Polymers: Poly(lactic-co-glycolic acid), Alginate** **Other materials:** -	Significant increase in total IgG and IgM antibodies No cytotoxicity in lung, kidney, and liver	[306]
*Pseudomonas aeruginosa*	**Polymer: Poly(lactic-co-glycolic acid), Alginate** **Other materials:** -	Significant decrease in the bacterial burden in the spleen Considerably increased opsonic activity	[307]
*Pseudomonas aeruginosa*	**Polymer: Polyhydroxyalkanoate** **Other materials:** Selected epitopes	Induced the production of functional antibodies Lead to opsonophagocytic hilling Induced an overall serotype-independent immune response	[308]
*Escherichia coli*	**Polymer: Chitosan, Dextran sulfate** **Other materials:** Vitamin E, IutA protein	Improved formulation stability Controlled release of the associated antigen Higher IgG levels than in an alum-adjuvanted vaccine Stable formulation at room temperature for at least 3 months	[309]

## Data Availability

No new data were created or analyzed in this study. Data sharing is not applicable to this article.
